# Socioeconomic status (SES) as a determinant of adherence to treatment in HIV infected patients: a systematic review of the literature

**DOI:** 10.1186/1742-4690-5-13

**Published:** 2008-02-01

**Authors:** Matthew E Falagas, Efstathia A Zarkadoulia, Paraskevi A Pliatsika, George Panos

**Affiliations:** 1 Alfa Institute of Biomedical Sciences (AIBS), Athens, Greece; 2 Department of Medicine, Tufts University School of Medicine, Boston, Massachusetts, USA; 3 HIV unit, 1^st ^IKA Hospital, Athens, Greece

## Abstract

**Objectives:**

It has been shown that socioeconomic status (SES) is associated with adherence to treatment of patients with several chronic diseases. However, there is a controversy regarding the impact of SES on adherence among patients with the human immunodeficiency virus (HIV) infection or acquired immunodeficiency syndrome (AIDS). Thus, we sought to perform a systematic review of the evidence regarding the association of SES with adherence to treatment of patients with HIV/AIDS.

**Methods:**

We searched the PubMed database to identify studies concerning SES and HIV/AIDS and collected data regarding the association between various determinants of SES (income, education, occupation) and adherence.

**Findings:**

We initially identified 116 potentially relevant articles and reviewed in detail 17 original studies, which contained data that were helpful in evaluating the association between SES and adherence to treatment of patients with HIV/AIDS. No original research study has specifically focused on the possible association between SES and adherence to treatment of patients with HIV/AIDS. Among the reviewed studies that examined the impact of income and education on adherence to antiretroviral treatment, only half and less than a third, respectively, found a statistically significant association between these main determinants of SES and adherence of patients infected with HIV/AIDS.

**Conclusion:**

Our systematic review of the available evidence does not provide conclusive support for existence of a clear association between SES and adherence among patients infected with HIV/AIDS. There seemed to be a positive trend among components of SES (income, education, occupation) and adherence to antiretroviral treatment in many of the reviewed studies, however most of the studies did not establish a statistically significant association between determinants of SES and adherence.

## Introduction

Suboptimal adherence to medical treatment with antiretroviral agents has been associated with increased morbidity and mortality, potential transmission of drug-resistant virus, drug resistance, and failure to achieve viral suppression [1-4]. Adherence to treatment in patients infected with the human immunodeficiency virus (HIV) or acquired immunodeficiency syndrome (AIDS) is influenced by factors associated with the patient, the disease, the patient-physician relationship, and the therapy [1-5]. Patient related determinants are socioeconomic status (SES), demographic, psychological, cognitive and behavioral characteristics [1,6-9].

It is suggested that SES is consistently associated with higher adherence to medical treatment in patients suffering from chronic diseases, such as asthma, diabetes, and post-myocardial infarction [1,7,10-12]. Suggested pathways in which SES might be associated with adherence, as well as morbidity and mortality, include education's effect on shaping a financially stable future, and on acquiring health literacy and knowledge to use health resources, while income plays a big part in obtaining better housing conditions, recreational facilities and better health care [13]. Moreover, occupation in terms of employment status affects the ongoing stress of the patients and their ability to use health care facilities, while occupational status can be reflected on the physical (possible environmental exposure to damaging agents) and psychosocial (lack of control over one's daily program) aspects of a low-SES patient's life [13]. All of these parameters influence accessibility to appropriate treatment and the patients' will to comply.

Although adherence is higher in patients with HIV/AIDS than in other chronic diseases (cardiovascular, infectious and pulmonary diseases) [7,14], it is not clear whether SES is associated with higher adherence to HIV therapy. A possible association between SES and adherence to treatment among HIV patients may have an impact on the success of their treatment, mainly because the knowledge of such an association may help the treating physicians identify patients who are less likely to adhere to treatment and thus, make more effort to influence the patient's adherence to treatment. In such a fashion, SES could affect the patient's quality of life, the social life of the patients and their families, the patient-physician relationship, and create a need for changes in matters of the public health system [1-4]. Subsequently, the effect of SES on adherence among HIV infected patients is considered a controversial issue [1,15,16]. Following the lead of other chronic diseases (diabetes, asthma, coronary disease), we hypothesized that a possible positive association between level of SES and level of adherence to antiretroviral treatment could exist and, thus, would be presented in our reviewed studies.

It is noteworthy that despite the fact that SES is a commonly used term, it is rather difficult to define and measure it [17]. According to "The New Dictionary of Cultural Literacy"(3d Edition 2002), SES depends on a combination of variables including occupation, education, income, and place of residence [18]. In this review, we attempted to synthesize the data regarding the association between SES and adherence to treatment of patients with HIV/AIDS, using information reported on major determinants of SES, namely income, education, and occupation.

## Methods

### Literature search

Two independent reviewers performed the literature search, study selection, and data extraction. Disagreements between these reviewers were resolved in meetings of all authors. We performed a systematic search of the literature to identify reviews and original studies that reported data regarding the impact of SES on adherence in HIV/AIDS patients. The relevant studies were identified by the use of the PubMed database (articles written in English), published until 2006. In addition, we performed additional searches of various Internet resources on HIV/AIDS [2,9,17,18]. Also, we searched the relevant articles identified from the list of references of the initially retrieved papers. We used 3 different search strategies using the following key words: 1. Socioeconomic status AND (HIV OR AIDS) AND (compliance OR adherence), 2. (Compliance OR adherence) AND (HIV OR AIDS) AND determinants, 3. (AIDS OR HIV) AND (compliance OR adherence) AND education AND income.

### Study selection

The inclusion and exclusion criteria used for the studies reviewed, were set before the literature search. Studies included in our study concerned only individual HIV-infected adult patients and their adherence to antiretroviral treatment. Reviews and editorials were not included in our systematic review. We excluded studies focused on HIV prevention, quality of life, attitude, and health status of patients. We also excluded studies, which compared the outcomes of treatment with different antiretroviral drugs without reporting specific data for the SES of the studied patients. Additionally, we excluded studies that focused on HIV-infected illicit drug users, as such users have specific psychosocial characteristics [19] and are in need of a special approach in order to adhere to medical treatment [20], a fact that differentiates them from the general population.

### Data extraction

From the studies that were included in our systematic review we extracted data regarding the date of publication, the setting of the study, the patient population, details of the medical treatment (monotherapy, Highly Active Anti-Retroviral Therapy – HAART), data relevant to SES, the measure of adherence, the overall adherence and findings regarding the association between major determinants of SES and adherence. In this study we assessed three parameters as major factors contributing to SES, namely, income, education, and occupation, and we examined their association with adherence to treatment of HIV infected patients.

## Findings

**Figure 1:**
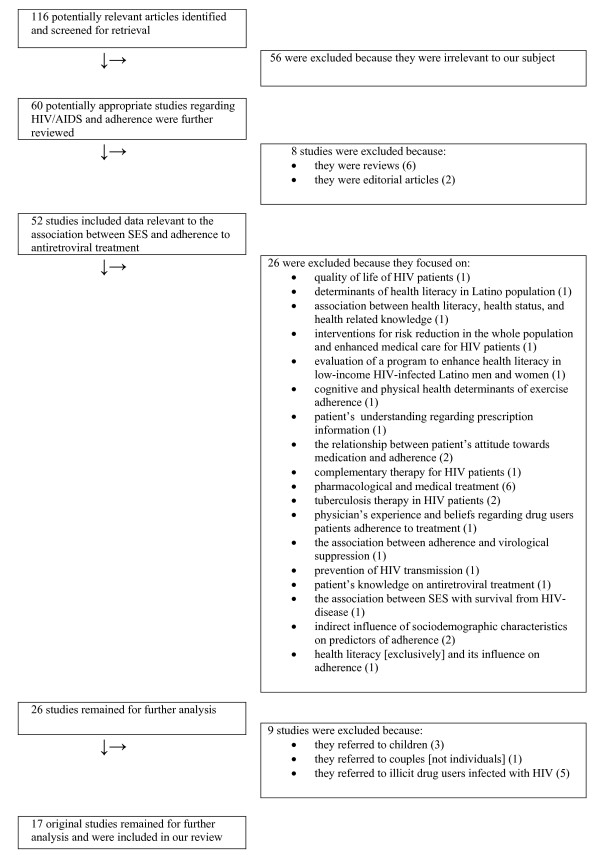
**Flow diagram of reviewed studies**. Flow diagram of all reviewed studies, showing how we ended up with the 17 original studies we further analyzed.

In Figure 1 we present the various steps in the study selection process. There were 116 potentially relevant studies from which we further reviewed 17 studies with original data. In Table 1 we present the characteristics of the 17 studies that were included in our systematic review. The year of publication of the studies ranged from 1991 to 2005. There was considerable variability among studies regarding the setting and the patient populations including different countries and different average socioeconomic and cultural background, respectively. In some studies the sample size of the population was small [4,21,22]. We reviewed 9 longitudinal [3,4,14,16,21-25] and 8 cross-sectional [15,26-32] studies, while the average patient number of the total 17 studies was 411 patients per study (ranging from 40 to 2267, depending on the study setting). The populations had previously been introduced to HAART in at least 12 of the reviewed studies [4,14-16,23-26,28-30,32]. Details regarding the antiretroviral treatment, such as the specific regimens used or the percentage of the population using them, were not reported in several studies [3,16,23,27,30,31]. Moreover studies varied in the measurement of adherence [pills per dose, doses per day, days of treatment per week(s), respect of the exact time schedule of obtaining the medications, etc] and used different cutoff point of adherence (from 80% to 100% of dosage) in order to dichotomize the patients between adherent and non-adherent.

**Table 1:** Design characteristics of the studies included in our systematic review

**First author, Year of publication [Reference number]**	**Setting**	**Type of Study**	**Patient Population**	Type of Medication (*)
Laniece I., 2003 [23]	Senegal, Dakar, 3 health structures	Prospective cohort study (2 years)	158 HIV(+) adults, enrolling into ISAARV (Senegalese ARV Access Initiative)	HAART, mainly
Mohammed H., 2004 [26]	USA, Non-urban Louisiana, 8 HIV outpatient clinics	Retrospective study (clinic survey) (30 months)	273 HIV(+) adults, using HAART	HAART
Eldred L.J., 1998 [27]	USA, Baltimore, Johns Hopkins Hospital, HIV Outpatient Clinic	Retrospective study (clinic survey) (9 months)	244 HIV(+) adults, Medicaid-insured, at least one previous clinic visit in previous 6 months + prescription of antiretroviral therapy for at least 6 months	Antiretroviral monotherapy, mainly
Kleeberger C.A., 2004 [24]	USA, Multicenter (4 centres in Baltimore, Chicago, Pittsburgh, Los Angeles)	Prospective cohort study (2 years)	597 HIV(+) homosexual men, using HAART + participating in MACS (Multicenter AIDS Cohort Study), between patients' 30^th ^and 33^rd ^visit [only 486 provided needed data on follow-up]	HAART
Peretti-Watel P., 2005 [28]	France, 102 hospital departments delivering HIV care	Cross-sectional study (national survey) (1 year)	1809 HIV(+) adults (homosexual men, heterosexual men, and heterosexual women), French speaking, diagnosed as HIV(+) for at least 12 months, living in France for at least 6 months + sexually active during the prior 12 months	HAART
Fong O.W., 2003 [15]	Hong Kong, Integrated Treatment Centre of the Department of Health	Retrospective study (1 year)	161 HIV(+) adults, Chinese in origin + treated with HAART for at least 12 months (at the end of 2000)	HAART
Kleeberger C.A., 2001 [25]	USA, Multicenter (4 centres in Baltimore, Chicago, Pittsburgh, Los Angeles)	Prospective cohort study (6 months)	539 HIV(+) homosexual men, during their 30^th ^visit to MACS	HAART, mainly
Goldman D.P., 2002 [16]	USA	Retrospective analysis of prospective study, (2 years)	2864 HIV(+) adults, participating in HCSUS (HIV Cost and Services Utilization Study [only 2267 provided needed data on last follow-up]	HAART, mainly
Golin C.E., 2002 [14]	USA, North Carolina, County Hospital HIV Clinic	Prospective cohort study (1 year)	117 HIV(+) adults, English or Spanish speaking + newly initiating HAART (PI or NNRTI)	HAART
Singh N., 1999 [3]	USA, 3 Medical Centres, HIV Clinics	Prospective cohort study (6 months)	123 HIV (+) adults, followed in any of the clinics	Antiretroviral treatment, not specified
Kalichman S.C., 1999 [29]	USA, Georgia, Atlanta, community area	Community-based study (Regional survey)	184 HIV(+) adults, receiving triple-drug combination	HAART
Weiser S., 2003 [30]	Botswana, 3 private clinics (2 in Gabarone, 1 in Francistown)	Cross-sectional study (Clinic survey) (7 months)	109 HIV (+) adults	Antiretroviral treatment (HAART 31%)
Morse E.V., 1991 [21]	USA, Louisiana, New Orleans	Nurse-based survey (6 months)	40 HIV (+) adults, asymptomatic + participating in ACTG (AIDS Clinical Trials Group) [the 20 most and the 20 least adherent patients]	ZDV or placebo
Gebo K.A., 2003 [31]	USA, Baltimore, Johns Hopkins University, HIV Clinic	Cross-sectional study (Clinic survey) (8 months)	196 HIV (+) adults, enrolling in the HIV Clinic + taking at least 1 antiretroviral medication	Antiretroviral treatment, not specified
Duong M., 2001 [32]	France, Dijon Hospital AIDS day-care Unit	Prospective cross-sectional study (5 months)	149 HIV (+) adults, receiving drug regimens including 2 nucleoside analogues + 1 or more PIs	HAART
Ickovics J.R, 2002 [4]	USA, Multicenter (21 collaborating units)	Prospective analysis of Randomised Controlled Trial (24 weeks)	93 HIV (+) adults, participating in ACTG (AIDS Clinical Trial Group) protocol 307	dT4+ DLV+IDV, ZDV+3TC+IDV, ZDV+DLV+IDV
Singh N., 1996 [22]	USA, Pittsburgh VA Medical Center	Prospective study (12 months)	46 HIV (+) male adults	ZDV only (78%), ZDV + ddI (13%), ddI only (8%)

(*) Abbreviations in medication: HAART = highly active antiretroviral treatment, ZDV = zidovudine, dT4 = stavudine, DLV = delaviridine, IDV = indinavir, 3TC = lamivudine

We did not identify a study focused directly on the association between SES or its main determinants analyzed as a group and adherence. In Table 2 we present the available reported data regarding factors contributing to SES, the method with which adherence to antiretroviral treatment was measured, and the overall adherence. In 11 out of 17 studies included in our review, self-report by the patients was the main measure of adherence to treatment [15,16,23-31]. The main parameters affecting SES (income, education, occupation) were not examined as a group comprising SES, but were rather regarded as demographic characteristics in most reviewed studies [14,24,25,28-31], therefore many studies lacked data concerning some of the parameters. There were insufficient data regarding income in 6 [15,16,21,24,30,32] and educational level in also 6 [15,16,24,27,28,31] of the 17 reviewed studies, respectively (some of the studies had data regarding income but not for education and others the reverse). Employment status was assessed in 9 studies [3,4,14,15,22,23,25,26,32], however no data were given on occupational status or working position. Health literacy was assessed in 1 study [29]. We considered this characteristic closely connected to educational level, therefore we included it as part of education in the presentation of the data.

**Table 2:** Socioeconomic characteristics and adherence measurement in the studies included in our review.

**First author, Year of publication [Reference number]**	**SES**			**Adherence**	
	
	**Income**	**Education**	**Employment**	**Measure of adherence**	**Adherence**
	
Laniece I., 2003 [23]	Median monthly income: 15,000 FCFA (about 20 US$) [80 (50.6%) participate in clinical trials and are free of charge]	Without school education: 50 (32%)	Not in paid employment: 65 (41%)	Self-reported number of tablets taken + number of tablets prescribed (by dispensing pharmacist), monthly. Mean and optimal (= 100% of dosage) adherence measured.	69% of self-reports optimal. 91% mean overall adherence self-reported.
Mohammed H., 2004 [26]	Monthly income: 0–999 US$: 220 (80.6%) >1,000 US$: 41 (15.0%) Missing 12 (4.4%)	High school or less: 184 (67.4%) Greater than high school: 79 (28.9%) Missing: 10 (3.7%)	Employed: 59 (21.6%) Unemployed: 205 (75.1%) Missing: 9 (3.3%)	Self-report of missing doses in previous week (interview with patient). Optimal (= 100% of dosage) adherence measured.	65.6% of self-reports optimal
Eldred L.J., 1998 [27]	Annual income: <$10,000 US$: 220 (91.3%) >$10,000 US$: 21 (8.7%) [All patients were insured and could cover treatment cost]	Grouped proportions not reported	No given data	Self-report of missing doses in previous week, self-report of missing days of treatment in previous 2 weeks (interview with patient) + examining medical record data of the Outpatient Clinic. Optimal (≥ 80% of doses and days) adherence measured.	Self-report vs. medical records: 60.4% vs. 55.8% optimal in previous week + 74.3% vs. 67.3% optimal in previous 2 weeks.
Kleeberger C.A., 2004 [24]	Grouped proportions not reported	Grouped proportions not reported	Grouped proportions not reported	Self-report of missing doses/pills in 4 previous days or not having a typical pattern in medication, every 6 months. Consecutive visit-pairs (1,128) were studied for decrease/increase in adherence from/to optimal to/from suboptimal. Optimal (= 100% of dosage) adherence measured.	88.7% of visit-pairs remained in optimal adherence. 71.5% of visit-pairs that reported suboptimal adherence in starting visit, increased to optimal in next visit. 38.8% of patients with 4 total visits reported suboptimal adherence, at least at one visit.
Peretti-Watel P., 2005 [28]	Financial situation of household satisfying: 1320 (73.0%) Housing conditions satisfying/acceptable: 1566 (86.6%) Food privation in household: 197 (10.9%)	No given data	No given data	Self-report of missing doses or not respecting time schedule, in previous week (interview with patient). Optimal (= 100% of dosage/timetable) adherence measured.	58% of self-reports optimal
Fong O.W., 2003 [15]	No given data	No given data	Busy workload: 16 (9.9%)	Self-report of missing doses since last follow-up, at each clinic visit Optimal (= 100% of dosage) adherence measured. Suboptimal adherence graded and measured.	80.7% of self-reports optimal. 15.5% of self-reports suboptimal but high grade of adherence (>95%). 1.9% of self-reports low grade of adherence (<90%).
Kleeberger C.A., 2001 [25]	Annual income: >50,000 US$: 165 (33.0%) <50,000 US$ 335: (67.0%)	College or more: 300 (56.3%) Less than college: 233 (43.7%)	Not full time: 178 (39.4%) Full time: 274 (60.6%)	Self-report of missing doses/pills in 4 previous days or not having a typical pattern in medication. Optimal (= 100% of dosage) adherence measured.	77.7% of self-reports optimal
Goldman D.P., 2002 [16]	No given data	Grouped proportions not reported	No given data	Self-report of missing doses/days of medication in previous week, on every follow-up. Optimal (= 100% of dosage) adherence measured.	Overall adherence not reported. 37.1%–57.3% optimal adherence to HAART, depending on years of schooling.
Golin C.E., 2002 [14]	Annual Income: <10,000 US$: 74 (63%) >10,000 US$: 43 (34%)	Less than high school: 41 (35%) High school or more: 76 (65%)	Working: 35 (30%) Not working: 82 (70%)	Evaluation of electronic medication bottle caps (MEMS) + pill count, every 4 weeks, and self-report of missing doses in the previous week, on 4 of the visits (interview with the patient). Mean and optimal (≥ 95% of dosage) adherence measured.	4% optimal adherence reported. 71% mean overall adherence reported.
Singh N., 1999 [3]	Monthly income: <500$: 22 (18%) 500–1,000$: 42 (34%) 1,000–1,500$: 27 (22%) >1,500$: 27 (22%) Not stated: 5 (4.1%)	Grade school: 5(4%) Technical: 6(5%) High school: 51(42%) College: 53(42%) Postgraduate: 8(7%)	Employed: 58 (47%) Unemployed: 65 (53%)	Refill methodology, monthly (all patients filled prescriptions exclusively through site pharmacy). Optimal (≥ 90% of dosage) adherence measured.	82% optimal adherence reported.
Kalichman S.C., 1999 [29]	<10,000 US$: 114 (62%) >10,000 US$: 70 (38%)	<12 years: 27 (14.7%) >12 years: 157 (85.3%) Lower health literacy TOFHLA: 29(15.8%)	No given data	Self-report of missing doses in previous 2 days (interview with patient). Mean and optimal (= 100% of dosage) adherence measured.	80.4% of self-reports optimal. 92.6% mean overall adherence self-reported.
Weiser S., 2003 [30]	No given data	Primary: 14 (13%) Secondary: 45 (41%) Post-secondary: 50 (46%)	No given data	Self-report of missing doses in previous day/week/month/year (interview with patient). Optimal (≥ 95%) adherence measured.	54% self-reports were optimal. An additional 29% of self-reports would be optimal if days of treatment hadn't been missed on financial grounds ('gaps in treatment').
Morse E.V., 1991 [21]	Proportion of patients receiving economic support by 'significant other' not reported	Less than high school: 2 (5.3%) High school graduates: 12 (31.6%) College: 10 (26.3%) College degree: 11 (29%) Professional/graduate degree: 3 (7.9%)	No given data	Nurse-based measurement of the Clinical Trial participants: 20 most adherent and 20 least adherent participants.	Not applicable.
Gebo K.A., 2003 [31]	Running out of money for life essentials in the previous 90 days: 104 (53%)	No given data	No given data	Self-report of missing doses in the previous 2 weeks (interview with patient). Mean and optimal (≥ 90% of dosage) adherence measured.	71% of self-reports optimal. 80% mean overall adherence self-reported.
Duong M., 2001 [32]	No given data	Grade school: 13 (9%) High school: 28 (19%) Technical school: 68 (46%) College: 40 (27%)	Employed: 80 (54%) Unemployed: 68 (46%)	Biological markers: HIV RNA undetectable or lower than criteria + PI plasma levels above reference. Optimal (= virologic response + adequate PI levels) adherence measured.	89% optimal adherence reported.
Ickovics J.R, 2002 [4]	Average yearly income: <$19,000: 47 (50.5%) >$20,000: 46 (49.5%)	High school or less: 39(42%) College/technical school or more: 54(56%)	Work for pay outside home: Yes: 67 (72%) No: 21 (23%) Missing: 5 (5%)	Self-report of number of pills skipped in previous 4 days (interview with the patient at baseline, week 2, week 4 and every 4 weeks thereafter through to week 24). Optimal (≥ 95% of dosage) adherence was measured.	63% of self-reports optimal.
Singh N., 1996 [22]	Median monthly income: 500–749 US$ No income: 5 (11%) >1,500 US$: 7 (15%) [All patients received treatment free of charge]	Less than high school: 10 (22%) High school: 9 (19%) College: 13 (28%) Technical education: 13 (28%) Postgraduate: 1 (2%)	Employed: 15 (33%)	Refill methodology, monthly (all patients filled prescriptions exclusively through site pharmacy). Optimal (≥ 80% of dosage) adherence was measured.	63% optimal adherence reported.

In Table 3 we present the main findings regarding the analysis of the association of the various components of SES and adherence. Income, level of education, and employment status were statistically significantly associated with the level of adherence in 7 [14,21,23,25,28,30,31], 5 [14,16,24,29,30], and 1 [15] original study, respectively (out of 17 studies reviewed); most significant findings refer to a positive association between levels of SES components and levels of adherence to antiretroviral treatment, although two of the reviewed studies suggest an adverse association between education [30] or having a busy workload [15], respectively, and adherence. However, the aforementioned SES determinants were not found to be statistically significantly associated with adherence in 7 [3,4,22,24,26,27,29], 8 [3,4,21,22,25-27,32], and 7 [3,4,14,22,24,25,32] other studies that examined such an association, respectively.

**Table 3:** Association between the main components of the socioeconomic status (SES) and adherence to treatment in HIV infected patients.

**First author, Year of publication [Reference Number]**	**Income**	**Education**	**Employment**	**Main Findings**
Laniece I., 2003 [23]	S.S.*	-*	-	Mean adherence among patients who were free of charge was higher than those participating in cost, in a statistically significant level, during 17 months of the study. Mean adherence among patients participating in cost + receiving D4T/ddI/IDV increased when cost participation decreased (during second year of study).
Mohammed H., 2004 [26]	N.S.*	N.S.	-	No SES components were significantly associated with adherence.
Eldred L.J., 1998 [27]	N.S.	N.S.	-	No SES components were significantly associated with adherence.
Kleeberger C.A., 2004 [24]	N.S.	S.S.	N.S.	Having less than a college education was an independent factor significantly associated with lowering adherence from optimal to suboptimal between two consecutive visits of the patient.
Peretti-Watel P., 2005 [28]	S.S.	-	-	Poor living conditions (except for food privation among homosexual men) were identified as an independent factor significantly associated with suboptimal adherence in all of the patients' subgroups.
Fong O.W., 2003 [15]	-	-	S.S.	Having a busy workload was found as an independent factor significantly associated with lower level of adherence.
Kleeberger C.A., 2001 [25]	S.S.	N.S.	N.S.	Annual income <50,000 US$ was identified as an independent factor significantly associated with lower level of adherence.
Goldman D.P., 2002 [16]	-	S.S.	-	Higher level of education was identified as a factor significantly associated with receiving HAART as a regimen and with higher level of adherence when using HAART.
Golin C.E., 2002 [14]	S.S.	S.S.	N.S.	Lower income and lower education were identified as independent factors significantly associated with lower level of adherence.
Singh N., 1999 [3]	N.S.	N.S.	N.S.	No SES components were significantly associated with adherence.
Kalichman S.C., 1999 [29]	N.S.	S.S.	-	Higher level of education and higher health literacy (among those with higher level of education) were identified as independent factors significantly associated with higher level of adherence.
Weiser S., 2003 [30]	S.S.	S.S.	-	Cost as a barrier to treatment was identified as an independent factor significantly associated with lower level of adherence (and gaps in treatment of otherwise would-be adherent patients). Incomplete secondary education was significantly associated with higher level of adherence.
Morse E.V., 1991 [21]	S.S.	N.S.	-	Receiving economic support by a 'significant other' was identified as an independent factor significantly associated with higher level of adherence.
Gebo K.A., 2003 [31]	S.S.	-	-	Running out of money for essentials during the previous 90 days was identified as an independent factor significantly associated with lower level of adherence.
Duong M., 2001 [32]	-	N.S.	N.S.	No SES components were significantly associated with adherence.
Ickovics J.R, 2002 [4]	N.S.	N.S.	N.S.	No SES components were significantly associated with adherence.
Singh N., 1996 [22]	N.S.	N.S.	N.S.	No SES components were significantly associated with adherence.

*S.S. = Statistically significant association found between SES component and adherence to treatment,

N.S. = No significant association found between SES component and adherence to treatment,

(-) = Association between SES component and adherence to treatment not examined

## Discussion

In this systematic review we found that SES was not consistently associated with adherence to treatment among HIV infected patients. Since there was no study directly examining the association between SES and adherence in patients with HIV/AIDS, we evaluated the available data regarding the possible association between the major separate determinants of SES (income, education, occupation) and adherence. Although someone would have expected a clear association between SES and adherence to treatment based on data from studies on patients with chronic diseases other than HIV/AIDS infection, the evidence from the available studies does not fully support the existence of such an association in this patient population. However, a positive trend of association between levels of various SES components and levels of adherence to antiretroviral treatment is present among many of the studies.

By taking a close look at the data presented, it is noteworthy that among the reviewed studies that examined some of the main components of SES, most did not find a statistically significant association between these factors and adherence to antiretroviral treatment. It should be emphasized that a statistically significant association between income and education, two main determinants of SES, and adherence was found in only half and less than a third of the studies that examined income and education, respectively.

The existence of a possible association between income and adherence to treatment in HIV/AIDS patients was examined in 14 of the reviewed studies. Among the 7 studies in which income was found to be significantly associated with adherence, 4 concluded that the cost of antiretroviral treatment and/or poor living conditions were factors preventing patients from complying with treatment. If this financial obstacle was overcome, adherence was expected to reach considerably higher levels [23,28,30,31]. In the remaining 3 studies, among patients having the economic ability to receive their medication, there was an association between the annual income and adherence [14,21,25]. It is presumed by the authors of one of the studies that patients with a higher level of income differ to those of lower/middle income, in terms of behavioral characteristics and hierarchy at the decision-making process, thus affecting their adherence to antiretroviral treatment [25]. Furthermore, perceived economic support by a significant other was found to have a direct association with levels of adherence to antiretroviral treatment, in another of the reviewed studies [21]. Such findings agree to the general idea linking stratification of income to disparities in health status and the will to adhere, placing the lower income patients on a deprivation scope, while allowing for higher income patients to adjust according to relative social status, possibly being influenced by other SES factors such as education and occupational status [13].

The existence of a possible association between level of education and adherence to treatment in HIV/AIDS patients was examined in 13 of the reviewed studies. Among the 13 studies that considered education as a probable factor affecting adherence to antiretroviral treatment, only 4 original studies [14,16,24,29] proved a statistically significant positive association. Education, providing the basis of a stable future for each person, as well as altering the criteria used during the decision-making process and the knowledge to access health resources and information on disease and treatment, is a powerful implement and could possibly be influenced by policies targeted to enhance adherence among HIV patients [5,6,16,29,33,34]. In 1 of the 4 studies, health literacy among those highly educated was also associated with higher level of adherence [29]. Health literacy is related to educational level, but is influenced by other determinants as well, such as health care providers' supportive manner and instructional skills [33], should therefore be considered a sector in which external intervention – and further training – is applicable [29,33,35]. Of note, in 1 of the 13 studies that examined the level of education, a statistically significant reverse association between this variable and adherence was found, although this interesting finding was not elaborated further by the authors of the reviewed study [30].

Employment status was either not assessed or not found to be an independent factor associated with adherence, in the majority of the studies that we reviewed. Specifically, employment was found to have a significant impact on adherence in only 1 of 8 studies that examined this factor. The authors of that study postulated that having a busy workload might be an impediment to the patients' ability to adhere to antiretroviral treatment [15], therefore suggesting an adverse association between adherence to antiretroviral treatment and a demanding working schedule. Unemployment and lower occupational status have, however, been linked to lower levels of health status and increased mortality [13] and could be blamed for lower levels of adherence in terms of stress caused by job insecurity, physical exhaustion, and lack of control over one's working schedule (as was the case in the reviewed study) [13,15], all of which could lead to a diminished intent and/or capability to follow antiretroviral treatment according to proper dosage and timetable [15]. We feel that further research should be carried out in order to estimate the possible effects of employment and occupational status on HIV patients' tendency to adhere to antiretroviral treatment.

Our systematic review has several limitations. First, it was not possible to make a synthesis of the data using the principles of meta-analysis due to the fact that there was considerable heterogeneity among the reviewed studies. Adherence was measured by different methods in each of the studies and the cutoff percentage of adherence to treatment between 'adherent' and 'non-adherent' varied among the studies, depending on the authors' estimate. Furthermore, while most of the studies included patients generally following the model of life prevailing in the industrialized countries, some of the studies focused on populations having special economic, cultural, and social structures. Moreover, the studied patients received different antiretroviral regimens, ranging from monotherapy to HAART; the complexity of the treatment schedule affects the level of a patient's adherence. Second, SES was not focused upon as a homogenous group of specific factors in any of the reviewed studies, but was rather dispersed among its components, which were regarded as socio-demographic information. Therefore, we were forced to collect partial data regarding the association of such SES components, and adherence to antiretroviral treatment, where – and if – such an association was assessed. Occupation was only assessed in terms of employment status, as no data were given on status of occupation or working position of the patients. Additionally, we could not analyze the possible association between other SES proxy variables, such as the neighborhood, and adherence to treatment because the included studies did not report relevant data. Third, patients supposed to have lower SES, as perceived by the treating physician, are generally more likely to receive less complex antiretroviral regimens, and more information on how to maintain a satisfying adherence level. We cannot exclude that such an inequity could have occurred in the reviewed studies, as most studies were not set in a randomized controlled trial (RCT) environment, and include random HIV patients, therefore impeding our effort to find an association between levels of SES, and adherence to antiretroviral treatment.

Adherence is a complex, dynamic process that influences the outcome of HIV treatment and the patient's health status [6,36]. It may change over time, as the health status or the patients' beliefs and attitude regarding the disease, the physician, and the treatment may alter, as well. As adherence does not concern only the patient, but the physician and the public health system too, it becomes evident that relevant factors cannot act independently, but instead they all interrelate [1,6]. Lower level of adherence to antiretroviral treatment leads to recurrence of the symptoms, drug resistance, and increases the patient's viral load, thus affecting the patient-physician relationship in a negative manner and creating possible hazards for the community, in terms of transmission, viral resistance, social stigma, and financial and/or management problems within the public health system [1-4]. Predicting patients that are expected to have lower adherence, in an objective manner, could establish an individual approach to secure each patient's optimal response to antiretroviral treatment, according to each patient's specific characteristics [5,31].

On the other hand, it has been noted before that physicians' choice regarding the medication they prescribe to their HIV patients is often influenced by their own estimates of expected level of patients' adherence to treatment, based on social stereotypes [5]. In this way, HIV patients with a low SES are less likely to be prescribed triple therapy [34,37]. However, the available evidence suggests that such estimates on expected patient adherence may have a limited accuracy and therefore should be treated with caution as they can result in harmful clinical consequences [30,36]. Also, the time the physicians devote to their patients and the methods they use in order to educate them about the HIV infection/disease, and convince them about the importance of adhering to treatment, depends on their judgments about the sociodemographic characteristics of the patients [5,36]. It is obvious that such an inequity in attention and instructions given by the physician, perhaps unavoidable in every day practice where patients gather in great numbers and time remains limited, results in uneven levels of co-operation and adherence between different patients.

Unlike SES, there were other factors, which were found to influence greatly and consistently HIV patients' adherence in the reviewed studies. Specifically, psychosocial factors such as depression [22,24,26,28,31], active drug [14,22,24,26,31] or alcohol use [14,26], and lack of social support and stability were associated with suboptimal level of adherence [2,3,5,8,21]. Furthermore, cognitive factors such as self-efficacy and patients' beliefs and views regarding the disease and the effectiveness of medication (outcome expectancies) were found to be significant determinants of adherence [3,4,14,27,32,38]. Also, adverse events were associated with lower level of adherence [4,8,30]. In general, complex schedule of drug therapy along with food restrictions were assessed as primary barriers to medication adherence [5,6,8,9,14,21,25,27]. The quality of the patient-physician relationship played an important role as well. Acceptance, open communication, cooperation and trust in physicians were reported to be strong predictors of enhanced adherence [1,2,5,6,21].

In several studies it has been shown that SES is significantly associated with adherence to treatment in patients with chronic diseases [10-12]. Despite the fact that HIV infection is included among chronic diseases, it differs from all others. This is probably due to the fact that this infection is socially stigmatized, in grounds of transmission. It is not only a physical disease, but a psychological, mental, and social, too. In addition, this infection is connected with social discrimination, guilt, and prejudice [5,28,30]. HIV infection is a life-changing event, affecting the psychological status of the patient and results in his/her having to adjust again, in new conditions of life. It seems that during this process, cognitive and psychological factors are more important than SES for adherence to therapy.

In order for HIV patients to achieve higher levels of adherence to treatment, interventions regarding the patient, the clinician and the treatment have to be made [5,6]. Specifically, helping patients to understand more about the HIV infection, as well as the antiretroviral treatment [5,6,16,29,33-35], coping with co-existing behavioral or psychiatric diseases [1,3,5,6], and adjust medication schedules to the patients daily program or using memory helpers such as special pillboxes, reminders etc. [5,6,14,15] are all important strategies. Additionally, the physician being consistent, vigilant, available, and explanatory can motivate the patient to adhere more to the antiretroviral treatment [1,38]. Warning the patients about potential side effects and coping with them timely, checking the list of medications at each visit, giving written information or showing pictures so as to provide instructions, are alternative and effective ways to ensure patients co-operation and participation in the therapeutic process [5,6,34]. As for the health system, it has to be noted that having a medical insurance and easy access to primary care, receiving treatment by the same medical providers each time, receiving counseling by specialists, and not having to pay for the antiretroviral regimens, are factors that enhance adherence level [2,4,5,9,21,30]. Improving a patient's financial and educational background is sometimes an impossible mission, however the aforementioned policies on educating and supporting the HIV patient can result in better adherence levels and should be investigated further, in terms of effectiveness.

Conclusively, the available evidence suggests that SES is not consistently associated with adherence to therapy among patients infected with HIV and it does not seem to be a major determinant of adherence to antiretroviral treatment. Many available studies suggest a positive trend among factors contributing to patients' SES and adherence to medical treatment among patients with HIV/AIDS, however such an association cannot be statistically consolidated throughout most of the studies included in our systematic review. It should be emphasized that it appears that there is a confusion regarding the accurate meaning of the term "SES" and thus it has been assessed in various ways. Future studies may further explain the different impact of SES to adherence to treatment between patients infected with HIV and patients suffering from other chronic diseases.

## Competing interests

The author(s) declare that they have no competing interests.
